# Malignant acanthosis nigricans associated with prostate cancer: a case report

**DOI:** 10.1186/1471-2490-14-88

**Published:** 2014-11-15

**Authors:** Joanna Kubicka-Wołkowska, Sylwia Dębska-Szmich, Maja Lisik-Habib, Marcin Noweta, Piotr Potemski

**Affiliations:** Department of Chemotherapy, Medical University of Lodz, Paderewskiego 4, 93-509 Lodz, Poland; Department of Dermatology, Paediatric Dermatology and Dermatological Oncology, Medical University of Lodz, Lodz, Poland

**Keywords:** Acanthosis nigricans, Prostate cancer, Paraneoplastic syndrome

## Abstract

**Background:**

Acanthosis nigricans is characterized by hyperpigmentation and hyperkeratosis of the skin or mucous membranes. Its malignant form is associated with internal neoplasms, especially gastric adenocarcinoma (55–61%). Coexistence with prostate cancer is uncommon. In the paraneoplastic type of this dermatosis, the skin and mucous lesions are characteristically of more sudden onset and more severe than those in the benign form. The efficacy of various treatment strategies remains disappointing.

**Case presentation:**

We here report a case of 66-year-old Caucasian patient with metastatic prostate cancer and a mild form of acanthosis nigricans that preceded the diagnosis of malignancy and resolved with chemotherapy in parallel with the prostate cancer. The dermatosis recurred when the prostate cancer progressed.

**Conclusion:**

Concurrent acanthosis nigricans and prostate cancer is rare, and few such cases have been reported. Anti-tumor therapy occasionally results in regression of this dermatosis. Underlying malignant disease should be suspected in individuals with elderly-onset of acanthosis nigricans.

## Background

Acanthosis nigricans (AN) manifests with hyperkeratotic areas with excessive pigmentation predominantly localized in the axillae, groin, on the nape of the neck or around the anus; the lesions less frequently affect oral mucous membranes. The diagnosis is based on the clinical characteristics of the lesions. AN can be classified into two forms: benign and malignant [1]. The first is more common and usually accompanies endocrinological disorders (type 2 diabetes, acromegaly, Cushing syndrome, hypothyroidism, and insulin resistance). It may also be congenital or produced by certain medications, including fusidic acid, nicotinic acid and certain hormones; in the latter, it characteristically regresses within 4–10 weeks of cessation of treatment [2]. A malignant form of AN (ANM) is associated with internal neoplasms, particularly gastric adenocarcinoma (55–61%) [3]; the associated neoplasms are less frequently located in the lungs, ovaries, breasts, kidneys, prostate or bladder [3–5]. The skin pathology may appear concurrently with the cancer (61%), as well as many years before (18%) or after its detection [6]. A coexisting neoplasm should always be suspected in patients with sudden onset of AN. Some reports have cited the presence of malignancy in almost 100% of cases of AN in elderly subjects [4]. Thus, a thorough evaluation for presence of malignancy is strongly recommended when AN is diagnosed in an elderly person.

The pathogenesis of AN is still widely debated; several possibilities are under consideration. Currently, it is presumed that certain cytokines, such as transforming growth factor-α (TGF-α), insulin-like growth factor 1 and fibroblast growth factor participate in the development of typical AN skin lesions. These tumor-produced substances stimulate keratinocytes, melanocytes and fibroblasts to proliferate. Many reports indicate that TGF-α plays a pivotal role in the development and progression of ANM [7–10]. In pathological conditions, excess TGF-α is produced and acts through epidermal growth factor receptors to induce hyperstimulation of keratinocytes. Ellis *et al*. and Koyama *et al*. independently reported resolution of skin lesions and decrease in urine and serum concentrations of TGF-α following surgical removal of malignant tumors in two patients [7, 8]. The factors responsible for development of skin lesions in patients with benign AN associated with endocrinopathies, obesity or diabetes are hyperinsulinemia and insulin resistance. Excessive proliferation and growth are induced particularly by insulin-like growth factor 1 [9, 11].

Herein we report a patient who developed ANM almost 1 year prior to the diagnosis of prostate cancer. Not only did complete remission of the skin lesions occur following administration of chemotherapy, progression of the underlying malignancy was accompanied by recurrence of AN.

### Case presentation

**Figure 1:**
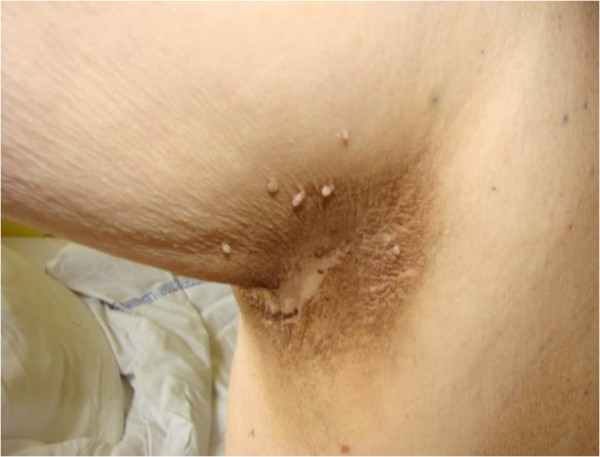
**Acanthosis nigricans before starting chemotherapy.** Right axilla

**Figure 2:**
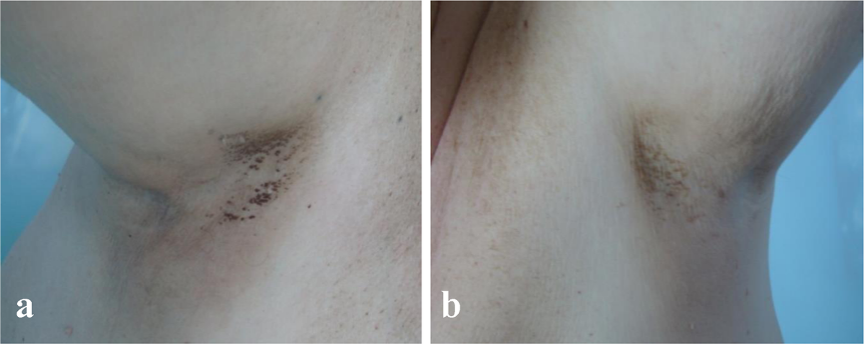
**Regression of skin lesions after five cycles of chemotherapy.** Right **(a)** and left **(b)** axilla.

**Figure 3:**
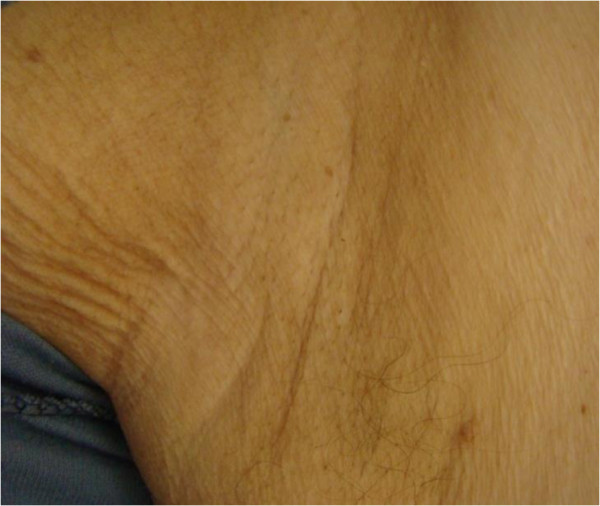
**Complete regression of skin condition (right axilla).**

**Figure 4:**
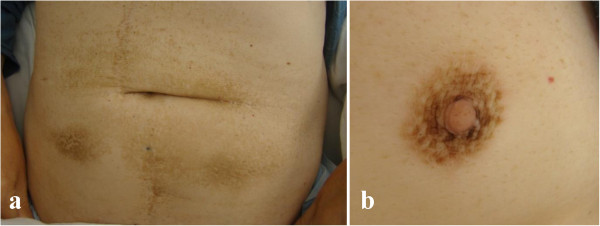
**Skin hyperpigmentation on the abdomen (a) and areola (b).** Recurrence of AN.

In December 2012, a 66-year-old patient with castration-resistant prostate cancer with bone metastases was admitted to the Chemotherapy Department, Medical University of Lodz to begin anti-neoplastic therapy. The patient had developed otherwise asymptomatic symmetric brownish skin discoloration affecting mainly his armpits in 2009. Because he had no associated symptoms, the family doctor he consulted had not started any therapy or performed any diagnostic tests. At the beginning of 2010, he was diagnosed as having type 2 diabetes and commenced oral antidiabetics, which were effective in controlling his diabetes. He had no history of other medical problems, endocrinopathies or dermatological disorders. Prostate cancer was diagnosed in February 2010 on biopsies taken because of a high prostate-specific antigen (PSA) serum concentration (13.56 ng/mL) and abnormal findings on digital rectal examination and trans-rectal ultrasonography. The neoplasm was classified as highly aggressive (Gleason score 4 + 4 = 8) and categorized as T2cN0M0 (IIB). The patient accordingly underwent bilateral pelvic lymphadenectomy and prostatectomy in the Urological and Transplantation Ward of Pirogow Memorial Hospital in Lodz. The final pathological evaluation revealed T3bN0 disease, positive resection margins and Gleason score 9 (4 + 5). In April 2011, bone scintigraphy revealed bone metastases and the patient began palliative hormonal therapy (luteinizing hormone-releasing hormone analog plus an antiandrogen). He is currently still receiving luteinizing hormone-releasing hormone analog injections. From June to October 2012, he received palliative radiation therapy to the pelvis, thoracic spine and right humerus for severe pain. Despite a good initial response to hormonal therapy, the patient’s prostate cancer eventually progressed and he was considered to have developed castration-resistant disease. In December 2012 he was referred to our Department for cytotoxic treatment. On admission, he was in good general condition (Eastern Cooperative Oncology Group performance status 1/2), his main complaint being of generalized bone pain. Dermatological evaluation revealed focal hyperpigmentation of the skin localized symmetrically in his axillae (Figure 1). The brownish skin discoloration was accompanied by verrucous-like lesions and mild pruritus. Mucous membranes and other areas of the skin were not affected. A diagnosis of AN was made based on the typical clinical appearance of his lesions. Aside from moderate grade gynecomastia probably attributable to hormonal treatment, there were no other relevant abnormalities on physical examination. Laboratory tests showed a castrate serum concentration of testosterone (<0.03 ng/mL) and increased concentrations of alkaline phosphatase (382 U/L), lactate dehydrogenase (269 U/L) and PSA (1280 ng/mL). Complete blood count revealed slightly decreased white blood cell count (4.19 × 103/μL), hemoglobin 11.5 g/dL, and hematocrit (35.1%). Electrolytes and other biochemical tests were within normal limits. After all clinical data had been collected, the patient was deemed eligible to receive standard cytotoxic treatment and accordingly received eight cycles of prednisone with docetaxel (75 mg/m^2^ every 3 weeks) from December 2012 to June 2013. Partial remission of skin lesions was apparent after five cycles of chemotherapy (Figure 2). After completion of the eight cycles, not only had his skin lesions completely regressed (Figure 3), but he also had reduced bone pain, a decrease in PSA serum concentration (162.6 ng/mL), improvement in his general well-being, and slight improvement in his bone metastases according to scintigraphy. In September 2013, recurrence of AN was noted in the form of non-itchy hyperpigmentation of the skin of the abdomen and areolae (Figure 4). There were no dermal lesions in the axillae. At the beginning of October, there was a sudden deterioration in the patient’s general condition with an increase in PSA concentration to 2852 ng/mL. Paraparesis caused by disease progression forced the patient to use a wheelchair. He has now received five cycles of second line chemotherapy with mitoxantrone and his prostate cancer is considered stable.

## Discussion

The initial skin lesions of ANM appear as areas of excessive keratosis and pigmentation that with time evolve into verrucous-like lesions. Unlike the benign form of AN, ANM occurs in the elderly (>40 years), has a rapid onset and spreads quickly, typically involving the mucous membranes or perianal area. Lesions in the oral cavity may involve the lips, gums, tongue, and palate and reportedly occur in 53% of patients [12]. In extreme cases, almost all of the skin is involved or sudden outbreaks of multiple seborrheic keratoses occur (Leser–Trelat sign). ANM is mainly associated with intra-abdominal malignancies (70–90%), especially gastric adenocarcinoma [3]. An association with prostate cancer is uncommon; to our knowledge, only a few such cases have been reported [3, 5, 13, 14]. Results of treatment aimed at relief of accompanying symptoms such as pruritus and pain in the oral cavity or anus, remain disappointing. However, the skin condition sometimes resolves with tumor-specific treatment (surgical resection, chemotherapy, radiotherapy), cyproheptadine, psoralen and ultraviolet A, or retinoids [15–19].

## Conclusions

An association between AN and prostate cancer is unusual. Reported cases of paraneoplastic AN, including those without severe skin lesions, emphasize the importance of vigilance concerning possible associated neoplasms, especially when the dermatosis has developed in an elderly individual. Even when the skin condition is relatively asymptomatic and perceived only as a cosmetic problem, it is essential to thoroughly investigate the possibility of neoplastic disease. Cooperation between urologists, oncologists and family doctors is crucial.

## Consent

Written informed consent was obtained from the patient for publication of this manuscript and accompanying images. A copy of the written consent is available for review by the Editor-in-Chief of this journal; however, the patient wishes his personal data to remain confidential.

## Abbreviations

AN: Acanthosis nigricans
ANM: Malignant acanthosis nigricans
TGF-α: Transforming growth factor alpha
PSA: Prostate-specific antigen.
